# P05 Pump and circumstance: a pilot study investigating the benefit of holding stock elastomeric infusers for the OPAT service at University Hospitals Birmingham

**DOI:** 10.1093/jacamr/dlaf239.009

**Published:** 2026-01-05

**Authors:** Marcus Mitchell, Nayab Hanif, Abigail Jenkins

**Affiliations:** University Hospitals Birmingham, Birmingham, UK; University Hospitals Birmingham, Birmingham, UK; University Hospitals Birmingham, Birmingham, UK

## Abstract

**Background:**

Due to the high cost and short shelf life of elastomeric antibiotic infusers these devices are ordered at University Hospitals Birmingham (UHB) from external commercial suppliers with a turnaround time from ordering to delivery of 4–23 days (turnaround times from 2024–25). This lag time causes delays to discharge resulting in patients who are otherwise medically fit for discharge remaining unnecessarily in a hospital in-patient bed. Pre-prepared infusers have a short shelf-life with a unit cost of £120–170 per day. Stocks of these medicines have previously not been held by pharmacy due to the risk of expiry and therefore waste.

**Objectives:**

To explore whether pre-prepared infusers held at the Queen Elizabeth Hospital inpatient Pharmacy as stock could be used to bridge the lag time to pump acquisition. It is proposed that this intervention will reduce time from referral to discharge for Queen Elizabeth Hospital patients accepted by the Trust OPAT team over a 4 week pilot study starting 1 April 2025.

**Methods:**

In the pilot elastomeric infusers containing flucloxacillin 8g or piperacillin-tazobactam 18g were stocked. Infusers were ordered during a four-week period and allocated by the OPAT pharmacist to patients who were referred and accepted into the OPAT service.

**Results:**

Eleven patients received treatment using pre-prepared stock infusers during the pilot phase which bridged the gap to supply by commercial providers. All eleven patients in the pilot commenced treatment within 24 h of referral to OPAT saving 58 inpatient bed days or 14.5 bed days each week (based on turnaround times stated by the outsourced suppliers at the time of the study). During the pilot phase 15 eligible patients at Queen Elizabeth were referred for early discharge, only eleven could be accepted due to the small-scale pilot. No infusers were wasted during this pilot period.

**Conclusions:**

Implementation of stock elastomeric infuser service at the Queen Elizabeth site of University Hospitals Birmingham facilitated patient flow which in turn offers potential savings due to a reduction in length of stay. Expanding the scale of future studies could evidence greater potential cost savings and the benefits when the demand is met for this service at UHB. Wider implementation of stock infusers to other UHB sites could offer the same reduced time to discharge for all OPAT infuser patients. Expansion to the holding of stock piperacillin/tazobactam 13.5 g elastomeric infusers and flucloxacillin 12 g elastomeric infusers at UHB could help improve patient flow and reduce hospital stays by enabling a wider range of eligible patients to receive elastomeric antibiotic pumps without acquisition lag time. However, further research is needed to evaluate the benefits of stocking these specific strengths in relation to the overall demand for them.

